# Dynamically controllable plasmon induced transparency based on hybrid metal-graphene metamaterials

**DOI:** 10.1038/s41598-017-14328-6

**Published:** 2017-10-24

**Authors:** Xicheng Yan, Tao Wang, Shuyuan Xiao, Tingting Liu, Haowen Hou, Le Cheng, Xiaoyun Jiang

**Affiliations:** 1 Wuhan National Laboratory for Optoelectronics, Huazhong University of Science and Technology, Wuhan, 430074 China; 2 School of Electronic Information and Communications, Huazhong University of Science and Technology, Wuhan, 430074 China; 3 Department of Electrical and Computer Engineering, National University of Singapore, Block E4, Engineering Drive 3, 117583 Singapore, Singapore

## Abstract

Novel hybrid metal-graphene metamaterials featuring dynamically controllable single, double and multiple plasmon induced transparency (PIT) windows are numerically explored in the terahertz (THz) regime. The designed plasmonic metamaterials composed of a strip and a ring with graphene integration generate a novel PIT window. Once the ring is divided into pairs of asymmetrical arcs, double PIT windows both with the spectral contrast ratio 100% are obtained, where one originates from the destructive interference between bright-dark modes, and the other is based on the interaction of bright-bright modes. Just because the double PIT windows are induced by two different mechanisms, the continuously controllable conductivity and damping of graphene are employed to appropriately interpret the high tunability in double transparency peaks at the resonant frequency, respectively. Moreover, multiple PIT windows can be achieved by introducing an additional bright mode to form the other bright-bright modes coupling. At the PIT transparent windows, the dispersions undergo tremendous modifications and the group delays reach up to 43 ps, 22 ps, and 25 ps, correspondingly. Our results suggest the existence of strong interaction between the monolayer graphene layer and metal-based resonant plasmonic metamaterials, which may hold widely applications in filters, modulators, switching, sensors and optical buffers.

## Introduction

Electromagnetically induced transparency (EIT) effect, which is produced by the quantum destructive interference between the two excitation pathways in laser-activated multiple level atomic systems^1^, has shown enormous potentials in the field of optical data storage^2^ and nonlinear optical enhancement^3^. However, rigorous conditions for the realization of conventional EIT effect have significantly limited the practical applications. Similar to the EIT effect, plasmon induced transparency (PIT), which is strong interaction among the bright-dark modes^2,4,5^ or bright-bright modes^6–9^, has been proposed in various plasmonic platforms such as plasmonic devices^10,11^, planar metamaterials^4,9,12–14^ and large area hybrid plasmon-waveguide systems^15^. Among different plasmonic systems, planar metamaterials become the research hot spot for the negligible propagation loss.

Graphene has been regarded as a new class of promising plasmonic material in photonics and optoelectronics due to many novel optical properties. At mid-infrared frequencies and terahertz (THz) frequencies, graphene has manifested a lot of unprecedented advantages including low propagation loss^16^, tight field confinement^17,18^, and the gate-voltage dependent feature^19–21^. Therefore, an effective route for control and manipulation of graphene-based dynamically controllable PIT effect has been proposed and the majority of these researches focus on the shifting of the whole PIT window accompanied by changing the Fermi energy of graphene^22–25^. For some applications, such as frequency-selective filter, it may be highly desirable to control specific transmission intensity while keeping the resonant frequency fixed. Recently, there have been many efforts in studying tunable metamaterials based on hybrid metal-graphene. Zhang *et al*. designed independently tunable perfect absorber based on hybrid metal-graphene metamaterials at mid-infrared frequencies^26^ and Sun *et al*. proposed independently tunable dual-band PIT based on hybrid metal-graphene^25^. Both of them extensively investigated the shift of the resonant frequency by changing the Fermi energy of the graphene. However, the intensity modulation of PIT has been rarely reported and the key role of graphene in the hybrid metal-graphene metamaterials has not been fully understood. Hence, the intensity modulation of PIT and how graphene works remain to be studied in the hybrid metal-graphene metamaterials.

Motivated by the previous observations, we investigate the dynamically controllable single, double and multiple PIT effects based on hybrid metal-graphene metamaterials by varying the Fermi energy in graphene, which consist of a strip and a ring with graphene integration structure. Both the strip and the ring are served as bright modes, which are induced by electric dipole oscillations. A novel PIT window caused by the hybridization of bright-bright modes can be controlled because of the oscillations of the metallic constructions damped by the dissipation of the graphene layer. By dividing ring into pairs of asymmetrical arcs, the other PIT transparency window with the spectral contrast ratio 100% emerges due to the excitation of magnetic dipole resonance. The continuously controllable conductivity of graphene can also explain the dynamically controllable amplitude of this PIT. Then, by introducing an additional bright mode to form the other bright-bright modes coupling, multiple PIT windows can be achieved and can be effectively controlled with different modulation depth approximately 39%, 86%, and 89% when the Fermi energy of graphene increases from 0.0 eV to 0.8 eV. At the transparent windows, the tremendous changes in the dispersions and the group delays reach up to 43 ps, 22 ps, and 25 ps, correspondingly. Compared with previously PIT effects, our structured plasmonic metamaterials presented have high feasibility and easy modulation.

## Results and Discussions

**Figure 1:**
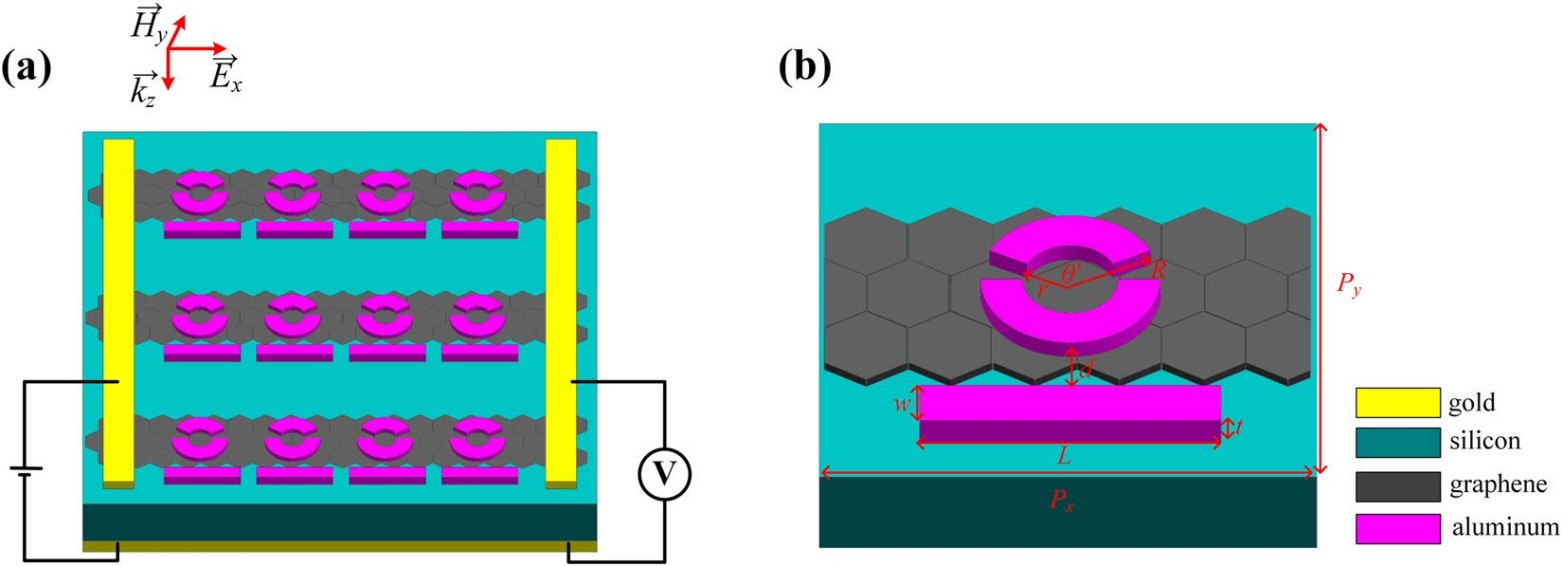
(**a**) Three–dimensional schematic of the hybrid metal-graphene metamaterials. (**b**) The unit cell of our proposed structure. The geometrical parameters are assumed to be *P*_*x*_ = *P*_*y*_ = 80 μm, *L* = 60 μm, *w* = 6 μm, *r* = 18 μm, *R* = 24 μm, *d* = 6 μm, *θ*′ = 155°, and *t* = 200 nm.

Figure 1(a) schematically shows our proposed plasmonic metamaterials, which consist of a strip and an asymmetric split ring (ASR) separated with a semi-infinite silicon substrate by a monolayer of graphene. All the graphene finger sets are connected to the bilateral gold strips as the top electrode and the lower gold square is used as the bottom electrode. And in general, similar high values of the Fermi energy in graphene can be observed with relatively low electric voltage applied^27^. Therefore, by applying the different gate voltages between the top electrode and the bottom electrode, we can achieve the flexibly controllable Fermi energy of graphene finger sets^28^. The unit cell is arranged in a two-dimensional array with a lattice constant *P*_*x*_ = *P*_*y*_ = 80 μm, which is illustrated in Fig. 1(b). The inner radius of the ASR is *r* = 18 μm with a ring width of 6 μm and spans different angles corresponding to *θ*′ = 155° and 180°. The strip placed 30 μm away from the center of the ASR has length *L* = 60 μm, width *w* = 6 μm. All metallic elements are chosen to be aluminum (Al) and have the same thickness *t* = 200 nm.

To our knowledge, the ASR supports electric dipole with currents flow symmetrically and magnetic dipole with currents flow antisymmetrically^29,30^. In the ASR, electric dipole, performed as the bright mode, is accessible from the free-space excitation with a broad transmission dip caused by the strong radiative losses, while magnetic dipole, regarded as the dark mode, cannot be directly coupled to the incident field because the magnetic arm of the incident plane wave is perpendicular to the array plane. However, the magnetic dipole can interact through near-field coupling with the bright mode, resulting in collective resonances of the ASR and the suppression of radiation damping^31,32^, forming the spectrally narrow dark mode.

**Figure 2:**
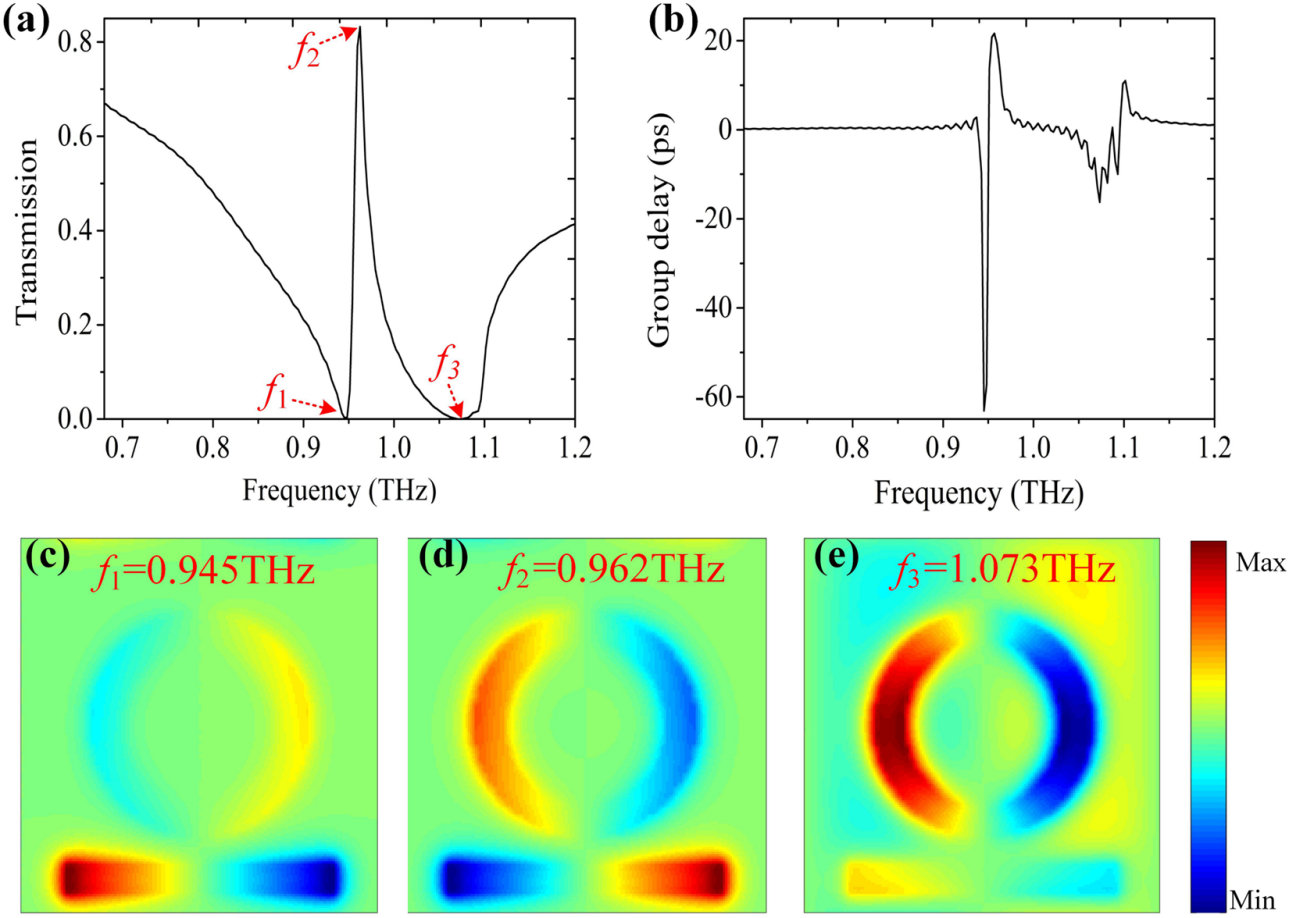
The simulated transmission spectra (**a**) and the calculated group delay (**b**) of the proposed metamaterials composed of a strip and a ring without the graphene layer. The geometric parameters are consistent with Fig. 1. (c)–(e) The electric field Ez distributions corresponding to the three feature points represented by *f*_1_, *f*_2_, and *f*_3_ in (**a**).

In the initial setup, we study the metamaterials composed of a strip and a ring without graphene integration. In all the simulations, a normally THz plane wave with x- polarization propagates along the z-axis and the geometric parameters are consistent with Fig. 1 except for *θ*′. As shown in Fig. 2(a), a PIT window with over 83% transmission at 0.962 THz located between two plasmon resonance dips at 0.945 THz and 1.073 THz. In order to fully understand the underlying physical mechanism behind the PIT effect, we present the simulated z-component of electric field (Ez) distributions in Fig. 2(c–e) corresponding to *f*_1_ = 0.945 THz, *f*_2_ = 0.962 THz, and *f*_3_ = 1.073 THz. From the Ez distributions at *f*_1_ = 0.945 THz (Fig. 2(c)), it is obvious that the strip is strongly excited by normal incidence light while the ring is scarcely excited. As for the other resonance dip at 1.073 THz (Fig. 2(e)), the strong interaction with exterior electric field primarily concentrates on the ring. Both plasmon resonance frequency at *f*_1_ and *f*_3_ serve as bright mode resonances. Notably, as a bright mode, the Ez distributions are not always observable at the plasmon resonance frequency *f*_1_ and *f*_3_ on account of the existence of the coupling field. In fact, it is electric dipole oscillations that form these bright modes. As shown in Fig. 2(d), we can see that both two parts are excited simultaneously due to the resonance detuning at *f*_2_ = 0.962 THz. The hybridization of the two parts leads to two almost identical but a antiphase quadrupole resonance, resulting in the PIT effect. It is worth reminding that the quadrupolar resonance can’t be excited by normal incidence light due to its vanishing dipole moment. Two different implementations are employed to activate the quadrupolar resonance: one is based on the highly angled illumination, but impractical, the other is based on the coupling with a bright dipolar resonance^6^. Thus, the quadrupole resonance mentioned above is exactly based on the latter in our proposed metamaterials.

Moreover, the most significant characteristic of the PIT effect is to achieve large positive group delay. Figure 2(b) provides the group delay, calculated by:

1 $${t}_{g}=\frac{d{\rm{\phi }}}{2\pi df}.$$

Here, ***φ*** is the phase shift introduced by the PIT effect. At the PIT window, there occurs a strong dispersion, giving rise to a large group delay, which means trapping photons for a long time inside our structure, and reaches up to 22 ps. The large positive group delay of our proposed metamaterials will have a better performance in the field of routing optical information^33^ and enhancing light-matter interactions^9^.

**Figure 3:**
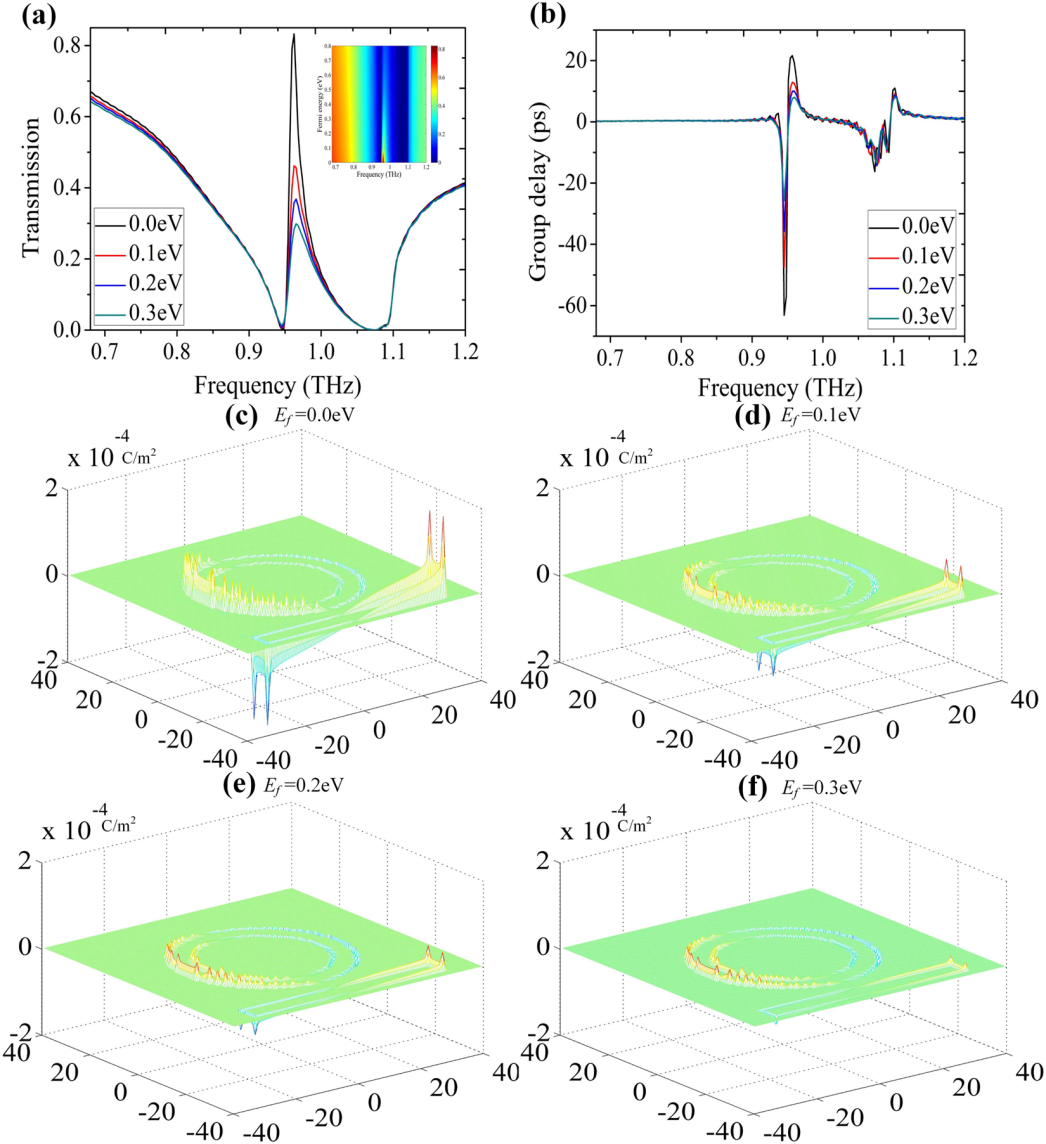
The simulated transmission spectra (**a**) and the calculated group delay (**b**) with different Fermi levels of graphene. The inset in (**a**) shows the evolution of the transmission spectrum versus Fermi energy and frequency. (**c**)–(**f**) The surface charge density distributions when the Fermi energy of graphene increases from 0.0 eV to 0.3 eV with a step of 0.1 eV.

We now add a monolayer graphene to the bottom of the ring to achieve dynamically controllable single PIT effect. In our current-in-plane device geometry, the work function of graphene is assumed to be equal everywhere by the use of a gate voltage to balance the effect of metal on the Fermi level^34–37^. The transmittance of the hybrid metal-graphene metamaterials with different Fermi energy of graphene is shown in Fig. 3(a). As the Fermi energy of graphene increases from 0.0 eV to 0.3 eV with a step of 0.1 eV, the transmission intensity of PIT window undergoes strong modulation, with transmission peak gradually shrinks at the fixed frequency, in the end, the PIT window drops distinctly. The change in this trend, is articulately illustrated in the inset of Fig. 3(a). In order to quantitatively describe the change of the transmission intensity with the graphene, the modulation depth in transmittance is defined as:

2 $${M}_{depth}=\frac{|(T-{T}_{g})|}{T}\times \mathrm{100 \% ,}$$

here *T* and *T*_*g*_ represent the transmission intensity without and with the graphene layer, respectively. When the Fermi energy of graphene is equal to 0.8 eV, the modulation depth *M*_*depth*_ of 83% at the frequency of 0.962 THz is realized. Therefore, the proposed metamaterials exhibit a high-performance optical switching property. During the modulation of PIT peak, we observe weakly dispersion, which will result in a significant decrease in group delay. We also calculate the group delay as shown in Fig. 3(b), the group delay declines from 22 ps to 8 ps when the Fermi energy of graphene increases from 0.0 eV to 0.3 eV. Our results may be useful for slow-light applications.

To explore the physical mechanism behind this novel phenomenon, the surface charge density distributions at the resonant frequency without (*E*_*f*_ = 0.0 eV) and with graphene at different Fermi levels are compared in Fig. 3(c–f). We observe that the surface charge distributions get more indistinctive when the Fermi energy of graphene increases from 0.0 eV to 0.3 eV. The physical mechanism lies in the additional dissipation caused by the resonant fields in the monolayer graphene, namely, the monolayer graphene can significantly damp the oscillation of plasmonic elements when it overlaps with the strong oscillation electric fields driven by the surface charge densities in the metallic elements. This phenomenon can also be quantitatively described as a dissipative power density^34^:

3 $$D={\sigma }_{g}{\Vert {E}_{res}\Vert }^{2},$$

here *σ*_*g*_ and *E*_*res*_ represent the conductivity of graphene and the resonant electric field, respectively. Due to the surface conductivity of graphene is relatively small, a big enough dissipative power density also demands that the resonant electric field is large enough. In the entire frequency range, only the frequency correspond to the PIT Peak has a large enough resonant electric field, thus, the transmission intensity of PIT peak undergoes strong modulation at the fixed frequency. When the resonant electric field is large enough at the resonant frequency, the enhancement of graphene conductivity via increasing its Fermi energy can strengthen the dissipative power density, as a result, the transmission intensity of PIT peak is substantially reduced and even turned off, which is in agreement with our trend presented above. The dynamically controllable PIT effect together with the physical mechanism can be strategically crucial in designing active hybrid metal-graphene metamaterials.

**Figure 4:**
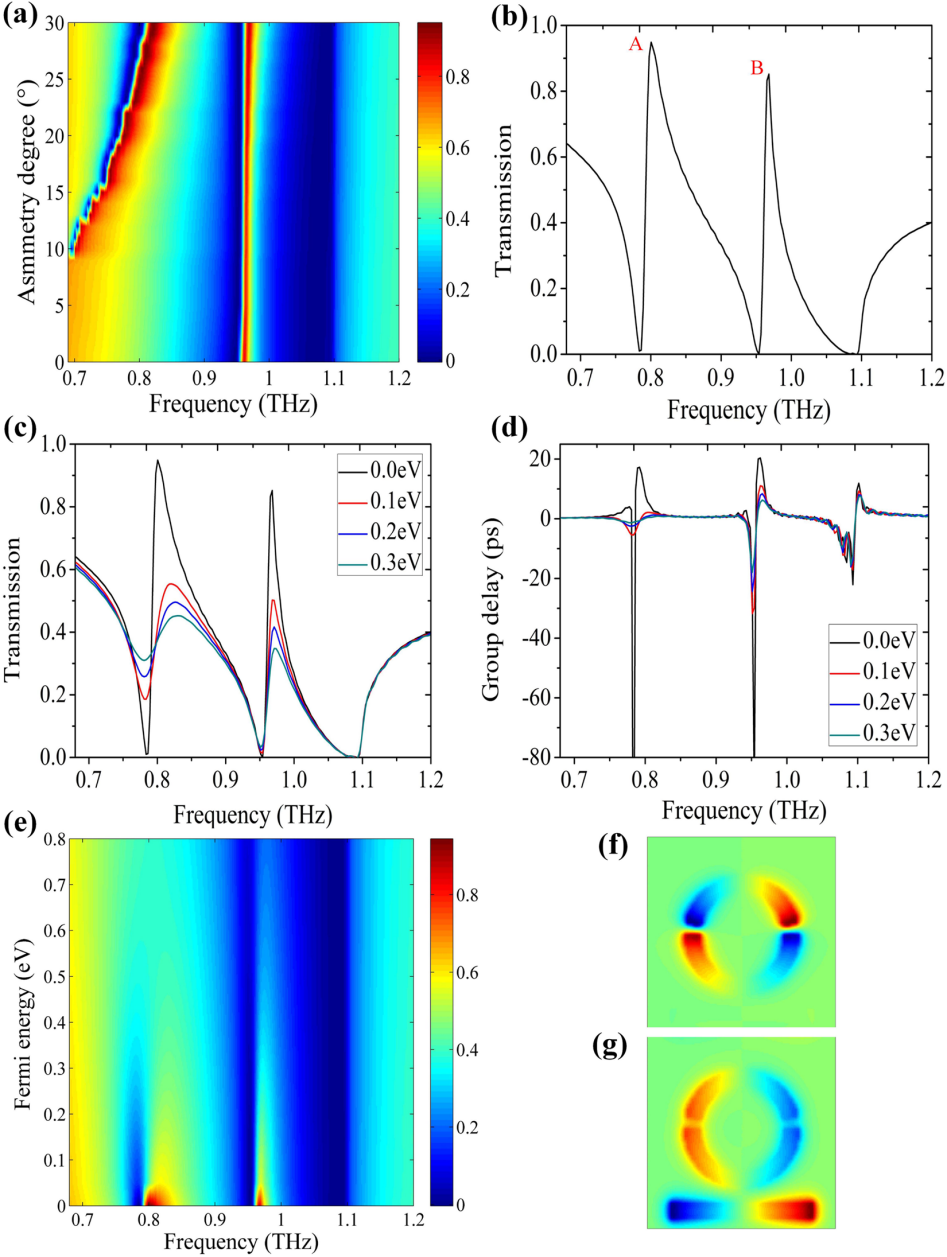
(**a**) The evolution of the transmission spectrum versus asymmetry degree and frequency. (**b**) The simulated transmission spectra of the proposed metamaterials composed of a strip and an ASR without the graphene layer. The simulated transmission spectra (**c**) and the calculated group delay (**d**) with different Fermi levels of graphene. (**e**) the evolution of the transmission spectrum versus Fermi energy and frequency. (**f**)–(**g**) Contour profiles of field Ez corresponding to the PIT peaks represented by A and B in (**b**).

It is widely recognized that the asymmetry degree in our proposed metamaterials plays an important role in optimizing the PIT window. So, the asymmetry degree is investigated with the decrease of *θ*′ and is defined as *θ* = 180−*θ*′. Figure 4(a) illustrates that an additional PIT window appears distinctly as the asymmetry degree gets larger on reason that the otherwise forbidden dark mode emerges in our proposed metamaterials. The additional PIT window grows in strength and has a blueshift as the asymmetry degree increases from 10° to 30° due to the decrease of effective length. In order to analyze the reason of the additional PIT window, we plot the simulated transmission spectra of the proposed metamaterials composed of a strip and ASR without the graphene layer as shown in Fig. 4(b), here, we select *θ* as 25°. Contour profiles of field Ez corresponding to the PIT peaks represented by A and B in Fig. 4(b) are also presented in Fig. 4(f) and (g). Apparently, the formation of the PIT B and the initial PIT discussed above have exactly the same mechanism, a result of quadrupole resonance. However, the occurrence of the additional PIT A is related to the destructive interference between the direct excitation of the electric dipole oscillation by the incident field in the strip and the excitation by coupling with magnetic dipole oscillation in the entire ASR. It should be noticed that the resonance frequency of the PIT channel A could be shifted by changing the asymmetry degree while the resonance frequency of PIT channel B is not affected. Thus, the operating frequency of optoelectronic devices based on the PIT (A) can be designed by altering the asymmetry degree of the metamaterials.

To evaluate the performance of the metamaterials in optical applications, the spectral contrast ratio of the PIT effect is introduced to be a key parameter, which is defined as:

4 $${S}_{con}=\frac{({T}_{peak}-{T}_{dip})}{({T}_{peak}+{T}_{dip})}\times \mathrm{100 \% ,}$$

where *T*_*peak*_ and *T*_*dip*_ represent the maximum transmission intensity and the minimum transmission intensity, respectively. As can be seen from Fig. 4(b), both the spectral contrast of the double PIT reach 100%. Thanks to the high spectral contrast ratio of the PIT, this proposed metamaterials authentically tend to exhibit as a promising candidate for the optical filtering, switch, and label-free sensing.

**Figure 5:**
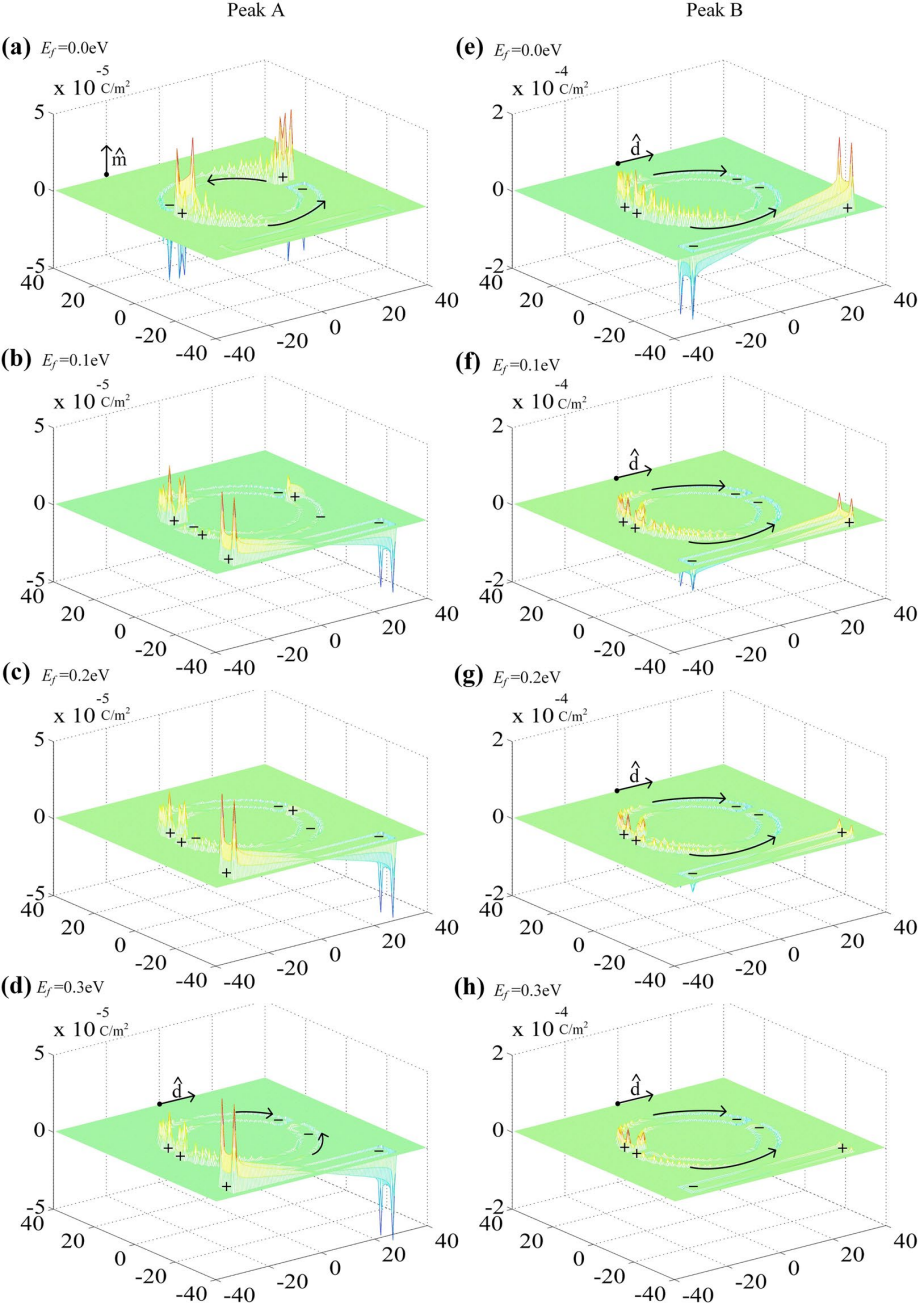
The calculated surface charge density distributions at PIT peak A (**a**)–(**d**) and Peak B (**e**)–(**h**) with the different Fermi energy of graphene. The asymmetry degree is fixed to 25° and the Fermi energy of graphene increases from 0.0 eV to 0.3 eV with a step of 0.1 eV.

Next let us consider the dynamically controllable double PIT windows in our proposed hybrid metal-graphene metamaterials. The simulated transmission spectra Fig. 4(c) and the calculated group delay Fig. 4(d) with different Fermi levels of graphene are presented. As the Fermi energy of graphene increases from 0.0 eV to 0.3 eV, the PIT peak A decreases from 95% to 45% with 53% modulation depth and the PIT peak B decreases from 85% to 33% with 61% modulation depth, corresponding to the group delay declines from 17 ps to 0 ps and 20 ps to 5 ps. Figure 4(e) is the evolution of the transmission spectrum versus Fermi energy and frequency. It is found that the double PIT windows undergo strong modulation and the modulation depths of 59% and 78% are achieved when the Fermi energy of graphene increases from 0.0 eV to 0.8 eV. According to the calculated surface charge density distributions in Fig. 5, we can syllabify the underlying physics. At the PIT A, a great quantity of the opposite kind of charges accumulate at each split gap, forming a circulation current along the entire ASR and generating a magnetic dipole along $\hat{m}$ as shown in Fig. 5(a). Therefore, the radiative losses of ASR are near completely suppressed. For the PIT B, a large amount of the same kind of charges locate at ends of each split gap, inferring a strictly symmetrical currents along the arcs and forming electric dipole along $\hat{d}$ as shown in Fig. 5(e). Once a monolayer graphene is placed on the bottom of the ASR, the opposite kind of charges are recombined and neutralized due to the shorting of the gaps by the conductive graphene. The magnetic dipole in the ASR gradually evolves into the electric dipole and the electric dipole in the strip was excited with the increase of the Fermi energy of graphene as shown in Fig. 5(a–d), which originates from the decline in the destructive interference between the electric dipole oscillation and magnetic dipole oscillation. However, the same kind of charges do not experience the charge redistribution and the surface charge distributions just get more indistinctive as the Fermi energy of graphene increases from 0.0 eV to 0.3 eV as shown in Fig. 5(e–h), which exhibits the same tendency with the previous discussion in the initial single PIT effet. Thus, this novel phenomenon is well explained by two different mechanisms. It is the continuously tunable conductivity and damping of graphene which lead to the dynamically controlled PIT A and B.

**Figure 6:**
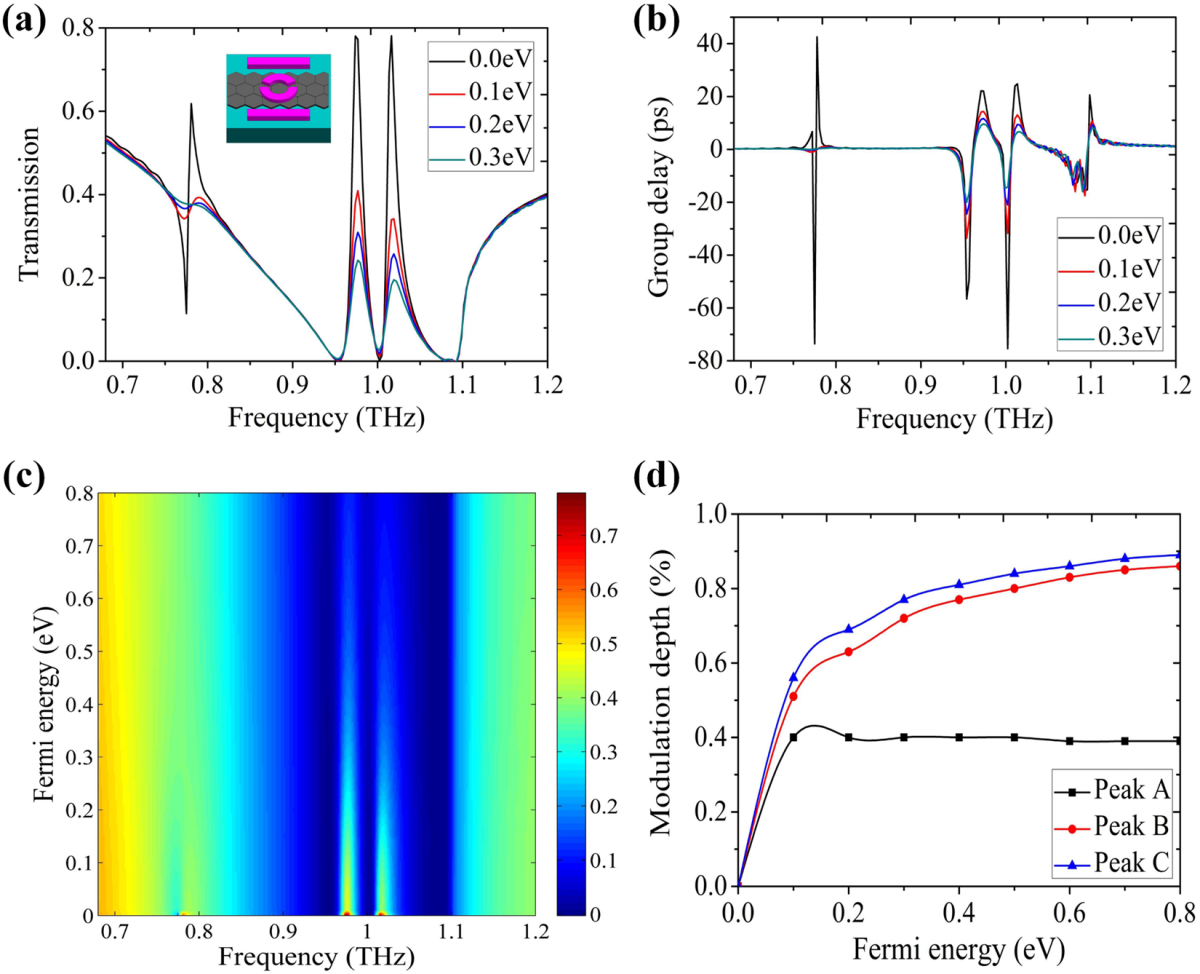
The simulated multiple PIT transmission spectra (**a**) and the calculated group delay (**b**) with different Fermi levels of graphene. The inset in (**a**) shows the unit cell of our proposed structure composed of symmetric double-strip and a ring with graphene integration. (**c**) the evolution of the transmission spectrum versus Fermi energy and frequency. (**d**) Modulation depth of Peak A/B/C against the Fermi energy of graphene.

Based on the previous structure comprising of a strip and ASR (*θ*′ = 25°) with the graphene layer, another strip regarded as bright mode is implemented to validate the multiple dynamically controllable PIT effects. The unit cell is shown in the inset of Fig. 6(a), which exhibits the simulated multiple PIT transmission responses with the spectral contrast ratio 95%, 100%, and 100%. When we change the double PIT metamaterials into the multiple PIT metamaterials, the transmission peak of PIT channel A decreases from 95% to 62% based on Fig. 4(c) and Fig. 6(a). The reason for this phenomenon is that the interaction between the incident plane wave and our designed structure becomes even greater. Compared with the results in Fig. 4(b), an extra PIT peak is obtained at the frequency of 1.016 THz, which is also caused by the hybridization of bright-bright modes. Obviously, when the Fermi energy of graphene increases from 0.0 eV to 0.3 eV, the transmission intensity of PIT channel A, channel B, and channel C varies from 62% to 38%, 78% to 11%, and 78% to 9%, correspondingly. Figure 6(c) shows the evolution of the observed multiple PIT effects versus the Fermi energy and frequency. It is found that as the Fermi energy increases the transmission intensity of PIT windows drops, and the modulation depth approximately 39% at 0.781 THz, 86% at 0.974 THz, and 89% at 1.016 THz is achieved when the Fermi energy of graphene increases from 0.0 eV to 0.8 eV. To understand the role of the Fermi energy in more detail, we present the modulation depth (Fig. 6(d)) of peak A/B/C against the Fermi energy of graphene, which once again confirms our proposed physical mechanisms. Finally, at the transparent windows, the phases experience continuous steep variation, leading to strong dispersions and tremendous group delays. As shown in Fig. 6(b), the group delays of the PIT transparency peaks are suppressed with the increase of the Fermi energy because of greater conductivity and dissipative power density. By dynamically tuning the Fermi energy of graphene, the group delay is controlled between 0.2 ps and 43 ps, 7 ps and 22 ps, and 5 ps and 25 ps, corresponding to the delay of a 60 μm and 12900 μm, 2100 μm and 6600 μm, and 1500 μm and 7500 μm distance of free space propagation. Therefore, demonstrating group delay control and profoundly characterizing the group delay spectrum, is a significant step toward the field of optical information routing and optical pulse trapping in the quantum information processing and optical communication.

## Conclusions

In conclusion, we propose dynamically controllable single, double and multiple PIT windows based on hybrid metal-graphene metamaterials consisting of a strip and a ring with graphene integration by tailoring the Fermi energy of graphene. The ring and strip are strongly excited by the incident field and are served as bright mode. The hybridization caused by the coupling of bright-bright modes generates a novel PIT window. When asymmetry is introduced to our structure, double PIT transparency windows originating from two different mechanisms are observed. One is based on the interaction between the electric dipole oscillation and magnetic dipole oscillation. And the other originates from the interaction between the electric dipole oscillation and the electric dipole oscillation. The continuously tunable conductivity and damping of graphene are employed to appropriately explain the controllable of double transparency amplitudes at the fixed frequency, respectively. Besides, multiple PIT windows are also predicted in our proposed plasmonic metamaterials, comprising a symmetric double-strip and a ring. More importantly, we can effectively control the intensities and group delays of the single, double and multiple transparency peaks with different modulation depth by tailoring the Fermi energy of graphene. These results will have widely applications in filters, modulators, switching, and sensors.

## Methods

We have calculated the results with the finite-difference time-domain (FDTD) method, using perfectly matched layer (PML) absorbing conditions in the propagation directions of the normally incidence plane wave and the periodical boundary conditions in the x and y directions to model the periodic array corresponding to the saturated array size. In the THz regime, the complex relative permittivity of aluminum *ε*_*Al*_ is given by the Drude model:

5 $${\varepsilon }_{Al}={\varepsilon }_{\infty }-\frac{{\omega }_{p}^{2}}{({\omega }^{2}+i\omega \gamma )},$$

where ${\varepsilon }_{\infty }$ = 1 is the dielectric constant at the infinite frequency, *γ* = 81.8 meV = 1.22 $\times {10}^{14}$ rad/s is the damping constant, and *ω*_*p*_ = 14.75 eV = 2.24 × 10^16^ rad/s is the plasmon frequency^38^. Also, we select semi-infinite silicon as substrate whose refractive index is *n*_*si*_ = 3.42.

**Figure 7:**
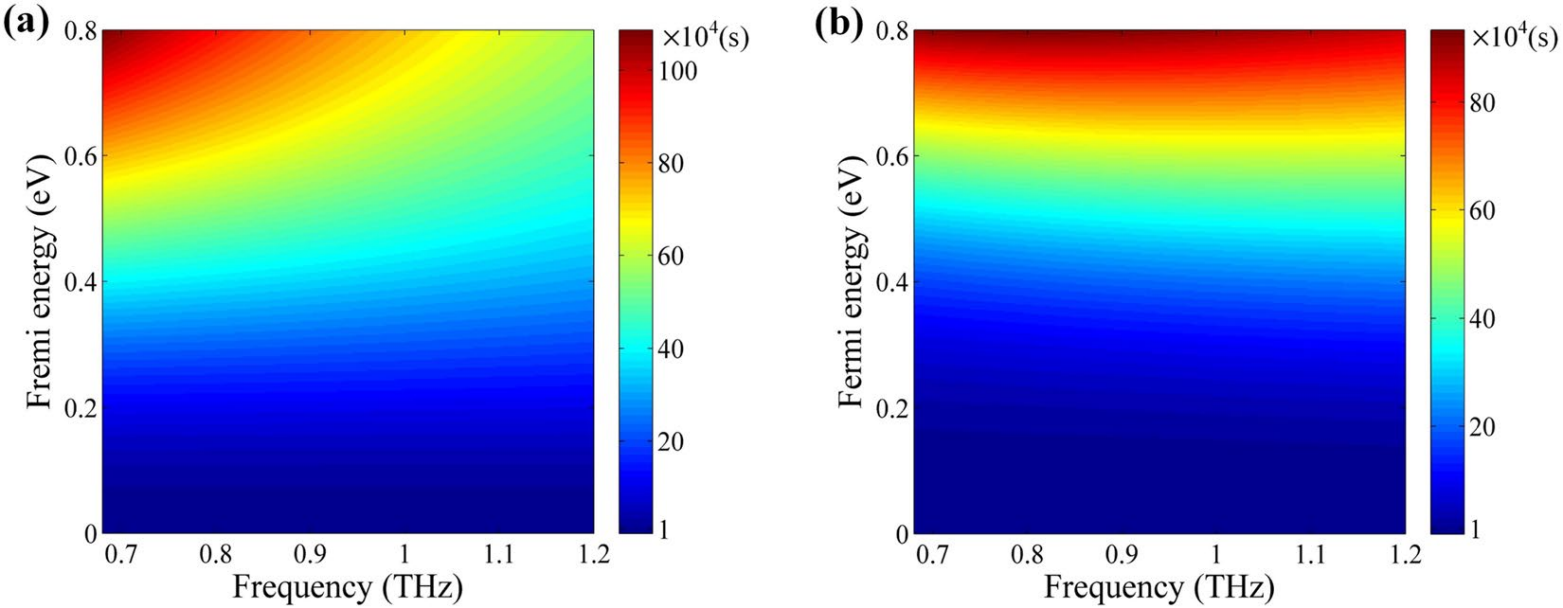
The real part (**a**) and imaginary part (**b**) frequency dependent surface conductivity of graphene corresponding to different Fermi energy.

Compared with the noble metal, graphene’s surface conductivity can be flexibly tailored by applying a gate voltage. In our simulations, graphene is modeled as a 2D flat plane whose conductivity presented by the Kubo formula^39^:

6 $${\sigma }_{g}=-j\frac{{e}^{2}{k}_{B}T}{\pi {\hslash }^{2}(\omega -2{\rm{\Gamma }}j)}[\frac{{E}_{f}}{{k}_{B}T}+2In({e}^{\frac{-{E}_{f}}{{k}_{B}T}}+1)]-\frac{{e}^{2}j}{4\pi \hslash }In(\frac{2|{E}_{f}|(\omega -2{\rm{\Gamma }}j)\hslash }{2|{E}_{f}|(\omega -2{\rm{\Gamma }}j)\hslash },)$$

where *e*, *k*_*B*_, *T*, *ћ*, Γ, *E*_*f*_, and *ω* is the electron charge, the Boltzmann’s constant, the temperature, the reduced Planck’s constant, the phenomenological scattering rate, the Fermi energy of graphene, and the angular frequency, respectively. The relaxation time $\tau =-1/(2{\rm{\Gamma }})=\mu {E}_{f}/(e{v}_{f})$ depends on the carrier mobility *μ*, the Fermi energy of graphene *E*_*f*_, and the Fermi velocity *v*_*f*_.. Notably, a high relaxation time in the graphene also reduces the transmission attenuation and means the difficulty in fabrication. So, we select the relaxation time *τ* = 70 fs, which consistent with the refs^40,41^. Figure 7 shows the real and imaginary part frequency dependent surface conductivity of graphene corresponding to different Fermi energy. It is clear that the increase of Fermi energy remarkable leads to an enhancement of the surface conductivity of graphene.

## References

[1] Boller K-J, Imamoğlu A, Harris SE (1991). Observation of electromagnetically induced transparency. Physical Review Letters.

[2] Kekatpure RD, Barnard ES, Cai W, Brongersma ML (2010). Phase-coupled plasmon-induced transparency. Physical Review Letters.

[3] Wu Y, Saldana J, Zhu Y (2003). Large enhancement of four-wave mixing by suppression of photon absorption from electromagnetically induced transparency. Physical Review A.

[4] Zhu, Y., Hu, X., Fu, Y., Yang, H. & Gong, Q. Ultralow-power and ultrafast all-optical tunable plasmon-induced transparency in metamaterials at optical communication range. *Scientific Rep* **3**, 2338 (2013). 10.1038/srep02338 PMC3730171 23903825

[5] Liu J-Q, Zhou Y-X, Li L, Wang P, Zayats AV (2015). Controlling plasmon-induced transparency of graphene metamolecules with external magnetic field. Optics Express.

[6] Jin X (2010). Plasmonic electromagnetically-induced transparency in symmetric structures. Optics Express.

[7] Dong Z-G (2010). Plasmonically induced transparent magnetic resonance in a metallic metamaterial composed of asymmetric double bars. Optics Express.

[8] Chen J (2011). Plasmonic eit-like switching in bright-dark-bright plasmon resonators. Optics Express.

[9] Wang J (2013). A novel planar metamaterial design for electromagnetically induced transparency and slow light. Optics Express.

[10] Zhu Y, Hu X, Yang H, Gong Q (2014). On-chip plasmon-induced transparency based on plasmonic coupled nanocavities. Scientific Rep.

[11] Chen J, Li Z, Yue S, Xiao J, Gong Q (2012). Plasmon-induced transparency in asymmetric t-shape single slit. Nano Letters.

[12] Fedotov VA, Rose M, Prosvirnin SL, Papasimakis N, Zheludev NI (2007). Sharp trapped-mode resonances in planar metamaterials with a broken structural symmetry. Physical Review Letters.

[13] Hokmabadi MP, Philip E, Rivera E, Kung P, Kim SM (2015). Plasmon-induced transparency by hybridizing concentric-twisted double split ring resonators. Scientific Rep.

[14] Liu G-q (2014). Robust multispectral transparency in continuous metal film structures via multiple near-field plasmon coupling by a finite-difference time-domain method. Physical Chemistry Chemical Physics.

[15] Zhang J (2011). Observation of ultra-narrow band plasmon induced transparency based on large-area hybrid plasmon-waveguide systems. Applied Physics Letters.

[16] Han X, Wang T, Li X, Xiao S, Zhu Y (2015). Dynamically tunable plasmon induced transparency in a graphene-based nanoribbon waveguide coupled with graphene rectangular resonators structure on sapphire substrate. Optics Express.

[17] Zeng C, Cui Y, Liu X (2015). Tunable multiple phase-coupled plasmon-induced transparencies in graphene metamaterials. Optics Express.

[18] Xiao S (2016). Tunable light trapping and absorption enhancement with graphene ring arrays. Physical Chemistry Chemical Physics.

[19] Liu J-Q, He M-D, Wang L-L (2013). Comment on “graphene metamaterial for optical reflection modulation”. Appl. Phys. Lett.

[20] Li, H., Wang, L. & Zhai, X. Tunable graphene-based mid-infrared plasmonic wide-angle narrowband perfect absorber. *Scientific Rep* **6**, 36651 (2016). 10.1038/srep36651 PMC5109233 27845350

[21] Xiao S (2017). Strong interaction between graphene layer and fano resonance in terahertz metamaterials. Journal of Physics D: Applied Physics.

[22] Shi X (2013). Plasmonic analog of electromagnetically induced transparency in nanostructure graphene. Optics Express.

[23] Xia S-X (2016). Dynamically tunable plasmonically induced transparency in sinusoidally curved and planar graphene layers. Optics Express.

[24] Yan, X. *et al*. High sensitivity nanoplasmonic sensor based on plasmon-induced transparency in a graphene nanoribbon waveguide coupled with detuned graphene square-nanoring resonators. *Plasmonics* **12**, 1449–1455 (2017).

[25] Sun C, Dong Z, Si J, Deng X (2017). Independently tunable dual-band plasmonically induced transparency based on hybrid metal-graphene metamaterials at mid-infrared frequencies. Optics Express.

[26] Zhang Y (2015). Independently tunable dual-band perfect absorber based on graphene at mid-infrared frequencies. Scientific Rep.

[27] Thind JK, Kumar M, Kaushik BK (2015). Electrical tuning of optical delay in graphene-based photonic crystal waveguide. IEEE Journal of Quantum Electronics.

[28] Gusynin V, Sharapov S, Carbotte J (2007). Sum rules for the optical and hall conductivity in graphene. Physical Review B.

[29] Jenkins SD, Ruostekoski J (2013). Metamaterial transparency induced by cooperative electromagnetic interactions. Physical Review Letters.

[30] Xiao S, Wang T, Liu Y, Han X, Yan X (2017). An ultrasensitive and multispectral refractive index sensor design based on quad-supercell metamaterials. Plasmonics.

[31] Liu N (2009). Plasmonic analogue of electromagnetically induced transparency at the drude damping limit. Nature Materials.

[32] Yang Y, Kravchenko II, Briggs DP, Valentine J (2014). All-dielectric metasurface analogue of electromagnetically induced transparency. Nature Communications.

[33] Schnorrberger U (2009). Electromagnetically induced transparency and light storage in an atomic mott insulator. Physical Review Letters.

[34] Zou Y, Tassin P, Koschny T, Soukoulis CM (2012). Interaction between graphene and metamaterials: split rings vs. wire pairs. Optics Express.

[35] Choi MS (2013). Controlled charge trapping by molybdenum disulphide and graphene in ultrathin heterostructured memory devices. Nature Communications.

[36] Yu Y-J (2009). Tuning the graphene work function by electric field effect. Nano Lett.

[37] Li Q (2016). Monolayer graphene sensing enabled by the strong fano-resonant metasurface. Nanoscale.

[38] Ordal MA, Bell RJ, Alexander RW, Long LL, Querry MR (1985). Optical properties of fourteen metals in the infrared and far infrared: Al, co, cu, au, fe, pb, mo, ni, pd, pt, ag, ti, v, and w. Applied Optics.

[39] Chen P-Y, Alù A (2011). Atomically thin surface cloak using graphene monolayers. ACS Nano.

[40] Zhang Y, Tan Y-W, Stormer HL, Kim P (2005). Experimental observation of the quantum hall effect and berry’s phase in graphene. Nature.

[41] Jnawali G, Rao Y, Yan H, Heinz TF (2013). Observation of a transient decrease in terahertz conductivity of single-layer graphene induced by ultrafast optical excitation. Nano Letters.

